# Assignment of a dubious gene cluster to melanin biosynthesis in the tomato fungal pathogen *Cladosporium fulvum*

**DOI:** 10.1371/journal.pone.0209600

**Published:** 2018-12-31

**Authors:** Scott A. Griffiths, Russell J. Cox, Elysa J. R. Overdijk, Carl H. Mesarich, Benedetta Saccomanno, Colin M. Lazarus, Pierre J. G. M. de Wit, Jérôme Collemare

**Affiliations:** 1 Fungal Natural Products, Westerdijk Fungal Biodiversity Institute, CT, Utrecht, The Netherlands; 2 Laboratory of Phytopathology, Wageningen University, Wageningen, The Netherlands; 3 Institut für Organische Chemie, Leibniz Universität Hannover, Hannover; 4 Laboratory of Cell Biology, Wageningen University, Wageningen, The Netherlands; 5 School of Biological Sciences, University of Bristol, Bristol, United Kingdom; University of Nebraska-Lincoln, UNITED STATES

## Abstract

Pigments and phytotoxins are crucial for the survival and spread of plant pathogenic fungi. The genome of the tomato biotrophic fungal pathogen *Cladosporium fulvum* contains a predicted gene cluster (*CfPKS1*, *CfPRF1*, *CfRDT1* and *CfTSF1*) that is syntenic with the characterized elsinochrome toxin gene cluster in the citrus pathogen *Elsinoë fawcettii*. However, a previous phylogenetic analysis suggested that CfPks1 might instead be involved in pigment production. Here, we report the characterization of the *CfPKS1* gene cluster to resolve this ambiguity. Activation of the regulator *CfTSF1* specifically induced the expression of *CfPKS1* and *CfRDT1*, but not of *CfPRF1*. These co-regulated genes that define the *CfPKS1* gene cluster are orthologous to genes involved in 1,3-dihydroxynaphthalene (DHN) melanin biosynthesis in other fungi. Heterologous expression of *CfPKS1* in *Aspergillus oryzae* yielded 1,3,6,8-tetrahydroxynaphthalene, a typical precursor of DHN melanin. *Δcfpks1* deletion mutants showed similar altered pigmentation to wild type treated with DHN melanin inhibitors. These mutants remained virulent on tomato, showing this gene cluster is not involved in pathogenicity. Altogether, our results showed that the *CfPKS1* gene cluster is involved in the production of DHN melanin and suggests that elsinochrome production in *E*. *fawcettii* likely involves another gene cluster.

## Introduction

Secondary metabolites (SMs) are compounds produced by microbes, plants and insects that are often repurposed as medicines and pesticides. Equally important are SMs with harmful effects, such as mycotoxins and pathogenicity factors that poison animals or promote crop diseases. The vast majority of fungal SMs with a clear biological role *in situ* are pathogenicity or virulence factors, also known as effector SMs, which are produced by plant pathogens during infection of their respective hosts [1,2]. Non-specific toxins synthesised by hemi-biotrophic and necrotrophic fungi are compounds that necrotise host tissues indiscriminately, whilst host-selective toxins (HSTs) only cause necrosis on plants expressing corresponding susceptibility genes, thereby determining host range [2].

1,8-dihydroxynaphthalene (DHN) melanin is a virulence SM for several plant and human fungal pathogens. DHN melanin is required for the penetration of rice leaves by *Magnaporthe oryzae*, a process mediated by appressoria, dome-shaped cells specialized in piercing the plant cuticle and cell wall [3]. Failure to melanise the fungal cell wall results in immature appressoria that cannot generate the turgor pressure required to penetrate host tissues [4,5]. Tricyclazole, pyroquilon and other commercial compounds that inhibit DHN melanin biosynthesis are highly effective at preventing rice blast [6–8]. The same role in plant penetration was reported in several other plant pathogens, including *Colletotrichum kahawae* and *Diplocarpon rosae*, pathogens of coffee berries and roses, respectively [9,10]. It has been suggested that DHN melanin is also a photodynamic virulence factor used by *Pseudocercospora fijiensis*, the causal agent of black Sigatoka disease, to generate toxic reactive oxygen species during infection of banana [11]. In addition to its role in virulence, DHN melanin provides tolerance to many kinds of abiotic stresses, including radiation and extreme temperatures [9,10]. Accordingly, DHN melanin production is often linked to the development of survival structures. For example, DHN melanin accumulates in the cell wall of conidia and sclerotia of the plant pathogen *Botrytis cinerea*, but it does not play a role in the virulence of this pathogen [12]. In the plant endophytic fungus *Pestalotiopsis fici*, DHN melanin was recently shown to be essential for the development of multicellular conidia [13].

Fungal DHN is produced through a polyketide pathway, which starts with a non-reducing polyketide synthase (nrPKS) [14,15]. The first stable intermediate, 1,3,6,8-tetrahydroxynapthalene (4THN), can be produced through three distinct biosynthetic routes. In Sordariomycetes such as *Colletotrichum lagenarium* [16,17], the nrPKS carries a bi-functional release domain that produces the hexaketide acetyl THN (ATHN) through Claisen ring closure, and then deacetylates ATHN to release the pentaketide 4THN [18]. The nrPKS in the Eurotiomycete fungus *Exophiala dermatitidis* also releases ATHN [19], but the deacetylation step is instead performed by the discrete hydrolase, YG1 [20]. In other Eurotiomycetes fungi such as *Aspergillus* and *Penicillium* species, the nrPKS is a heptaketide synthase that releases YWA1 [21–23], which is deacylated by the hydrolase AYG1 to produce 4THN [21,24,25]. In certain fungal species like *B*. *cinerea*, two nrPKSs, likely one synthase with a bi-functional release domain (BcPks12) and one hexaketide or heptaketide synthase (BcPks13), are involved in DHN melanin biosynthesis [12]. The subsequent enzymatic steps to convert 4THN to DHN are common to all fungal species; 4THN is first reduced to scytalone by a 4HNR reductase, then dehydrated to 1,3,8-trihydroxynapthalene (3THN) by the dehydratase SCD1 [15]. 3THN is reduced to vermelone by the reductase 3HNR, then dehydrated by SCD1 to yield DHN [15]. These reductases, especially 3HNR, are the target of tricyclazole [26]. Finally, DHN is polymerized into melanin by multicopper oxidases [27–30]. These different pathways have been invoked to explain the difference in pigmentation between brown-black fungi, including *C*. *lagenarium*, *M*. *oryzae* and *C*. *heterostrophus*, and blueish-green fungi like *A*. *fumigatus* that might polymerize YWA1 in addition to DHN [30]. The genes encoding enzymes involved in DHN melanin biosynthesis and polymerization are organized in a gene cluster in *A*. *fumigatus* [30], and *Penicillium marneffei* [31], but they are partially clustered in *Alternaria alternata* and *Cochliobolus heterostrophus* [32,33] and tend to be dispersed in other fungal genomes [15].

It must be noted that certain fungal species produce another kind of melanin that is synthesized from L-3,4-dihydroxyphenylalanine (DOPA) through the action of tyrosinases and laccases [34]. Although the DHN melanin genes can be present in fungal genomes, the DOPA melanin pathway is the major route employed by certain fungal species, as exemplified by the pine needle pathogen *Dothistroma septosporum* [35].

*Cladosporium fulvum* is a non-obligate, biotrophic fungus that causes tomato leaf mold disease. *C*. *fulvum* shows limited filamentous growth on *in vitro* media in the dark, forming small sporulating colonies. They exhibit a green-brown colour, which was linked to the production of the pigment cladofulvin [36]. *C*. *fulvum* colonies harbour a grey colour when cladofulvin is not produced, which is likely due to the production of another pigment [36]. *C*. *fulvum* is known to reproduce asexually only and production of cladofulvin is primarily observed in conidia [37]. Despite a high potential chemical diversity with 23 predicted-functional SM core genes [38,39], the pigment cladofulvin produced by the *claG* gene cluster remains the only detectable SM [36,40]. It was suggested and later shown that the repression of cladofulvin biosynthetic genes is required for biotrophic growth of *C*. *fulvum* [37,38]. *CfPKS1* is another nrPKS core gene that shows a similar expression profile during infection of tomato leaves, *i*.*e*. downregulation [38]. *CfPKS1* belongs to a predicted gene cluster containing genes that encode a prefoldin chaperone (*CfPRF1*), a reductase (*CfRDT1*) and a transcription factor (*CfTSF1*) [38]. Previous comparative genomic analyses indicated that the *CfPKS1* gene cluster is homologous to the *Elsinoë fawcettii* gene cluster responsible for elsinochrome production, a light-activated toxin involved in the virulence of this pathogen on citrus hosts [41,42]. However, the phylogeny of CfPks1 suggested that it is also closely related to nrPKSs involved in DHN melanin biosynthesis [38]. In another study, *CfPKS1* was strongly up-regulated in the *C*. *fulvum* deletion mutant *Δ*cf*wor1* during growth on agar [43]. The hyper-black appearance of *Δcfwor1* colonies and the absence of detectable SMs suggested that CfPks1 might be involved in the production of polymerized DHN melanin in *C*. *fulvum*. Such ambiguity between elsinochrome and DHN production remains unresolved.

Here, we report the functional characterization of the *CfPKS1* gene cluster by targeted gene deletion (*CfPKS1*), over-expression of the predicted local regulator (*CfTSF1*), and heterologous expression in *Aspergillus oryzae*. We provide chemical evidence of the pigment produced by this pathway and assessed the role of this compound in pathogenicity and biotrophic growth of *C*. *fulvum*.

## Results

### Definition of the *CfPKS1* gene cluster in *Cladosporium fulvum*

**Fig 1:**
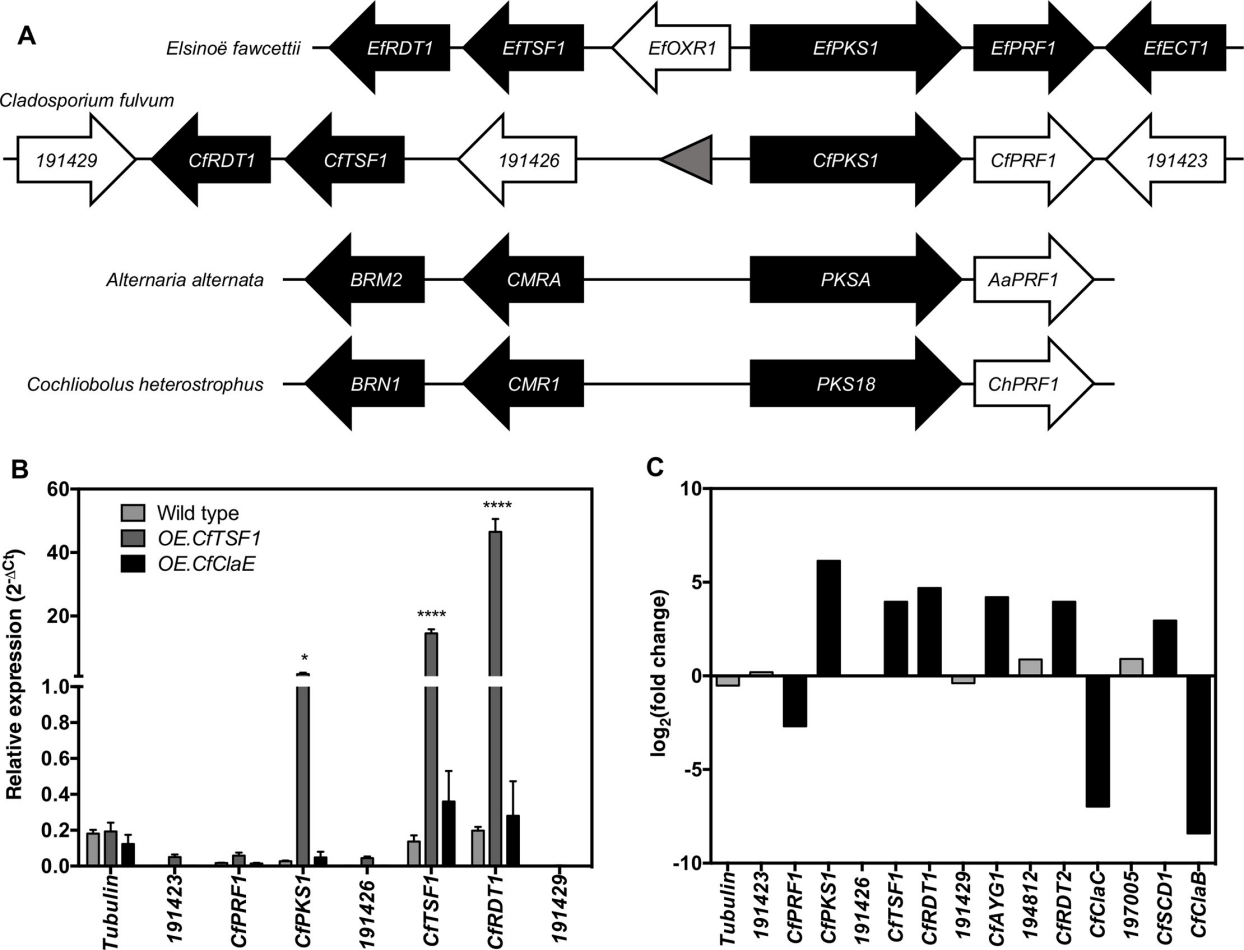
Definition of the *CfPKS1* gene cluster in *Cladosporium fulvum*. **(A)** Organization of the predicted *CfPKS1* gene cluster based on the reported homologous gene cluster in *Elsinoe fawcettii* [38,41]. The locus of the DHN melanin gene cluster in *Alternaria alternata* and *Cochliobolus heterostrophus* is shown for comparison [32,33]. All loci contain a prefoldin-encoding gene downstream of the polyketide synthase gene. *CMR1* and *CMRA* encode transcription factors that regulate the DHN melanin biosynthetic genes. *BRN1* and *BRM2* are 3-hydroxy naphthalene reductases homologous to *RDT1*. Black arrows indicate co-regulated genes in each species, white arrows indicate non-co-regulated genes and the grey triangle indicate a transposable element. Loci are not drawn to scale. **(B)** Relative expression of genes at the *CfPKS1* locus determined by reverse transcription-quantitative real-time polymerase chain reaction (RT-qrtPCR). Strain were pre-cultured in Potato Dextrose Broth (PDB) for five days before the biomass was transferred to B5 without nitrogen (B5-N) medium. After 48 hours, the biomass was recovered and used for RNA isolation and cDNA synthesis. The expression value for each gene within the *CfPKS1* locus was measured in wild-type *C*. *fulvum* and inducible over-expression tranformants *C*. *fulvum OE*.*CfTSF1* and *OE*.*CfClaE* [38] grown in B5-N medium. Expression values were normalised to *actin* and the average value was plotted with standard deviation between three biological replicates. A two-way ANOVA with a posthoc Sidak multicomparison test at the significance level of 0.05 was used to calculate statistically significant changes in gene expression between wild type and transformant strains. Asterisks (*) denote statistically significant changes (*p* < 0.05 or less). **(C)** Differential expression of genes at the *CfPKS1* locus and homologues of genes involved in the biosynthesis of DHN melanin in the *Δcfwor1* deletion mutant compared with the wild type as determined by RNA-seq [43]. Black and gray bars show significant and nonsignificant fold changes, respectively, according to Cuffdiff analysis of three biological repeats.

The *CfPKS1* gene cluster (Fig 1A) was initially predicted solely through its homology and synteny with the characterized elsinochrome gene cluster in *E*. *fawcettii*, as only minimal gene expression within this gene cluster had been observed during the growth of wild-type *C*. *fulvum* under diverse conditions [38]. The gene cluster includes *CfTSF1*, a gene predicted to encode a pathway-specific transcription factor [38,41]. To up-regulate and clearly define the *CfPKS1* gene cluster, wild-type *C*. *fulvum* was transformed with a plasmid containing *CfTSF1* fused to the promoter region of the nitrogen-regulated *C*. *fulvum Avr9* gene [44]. The resulting *OE*.*CfTSF1* transformant (S1 Fig) does not show any *in vitro* difference compared to wild type, but this transformant is not pathogenic on tomato (S2 Fig). Although random insertion of the expression cassette in a pathogenicity gene cannot be excluded, this loss of pathogenicity is likely due to the up-regulation of *CfTSF1* because the *Avr9* promoter induces high-expression when *C*. *fulvum* enters the plant and colonizes leaf tissues [38,44]. Both transformant and parental strain were grown in PDB and then induced in B5 medium without nitrogen (B5-N) for 48 hours to induce gene expression. Transcriptional profiling by RT-qrtPCR showed that the relative expression of *CfPKS1*, *CfTSF1* and *CfRDT1* was 1.7, 14.6 and 46.5-fold higher, respectively, in the *OE*.*CfTSF1* transformant than in wild type (each t-test P-value < 0.0001), whilst the predicted border genes are not co-regulated (Fig 1B). In contrast, *CfPRF1* was not co-regulated and is therefore unlikely part of the *CfPKS1* biosynthetic pathway. The up-regulation of the *CfPKS1* gene cluster is specific to the over-expression of *CfTSF1* because the gene cluster is not significantly activated when the regulator of cladofulvin production (*CfClaE*; 40) is over-expressed (Fig 1B).

**Fig 2:**
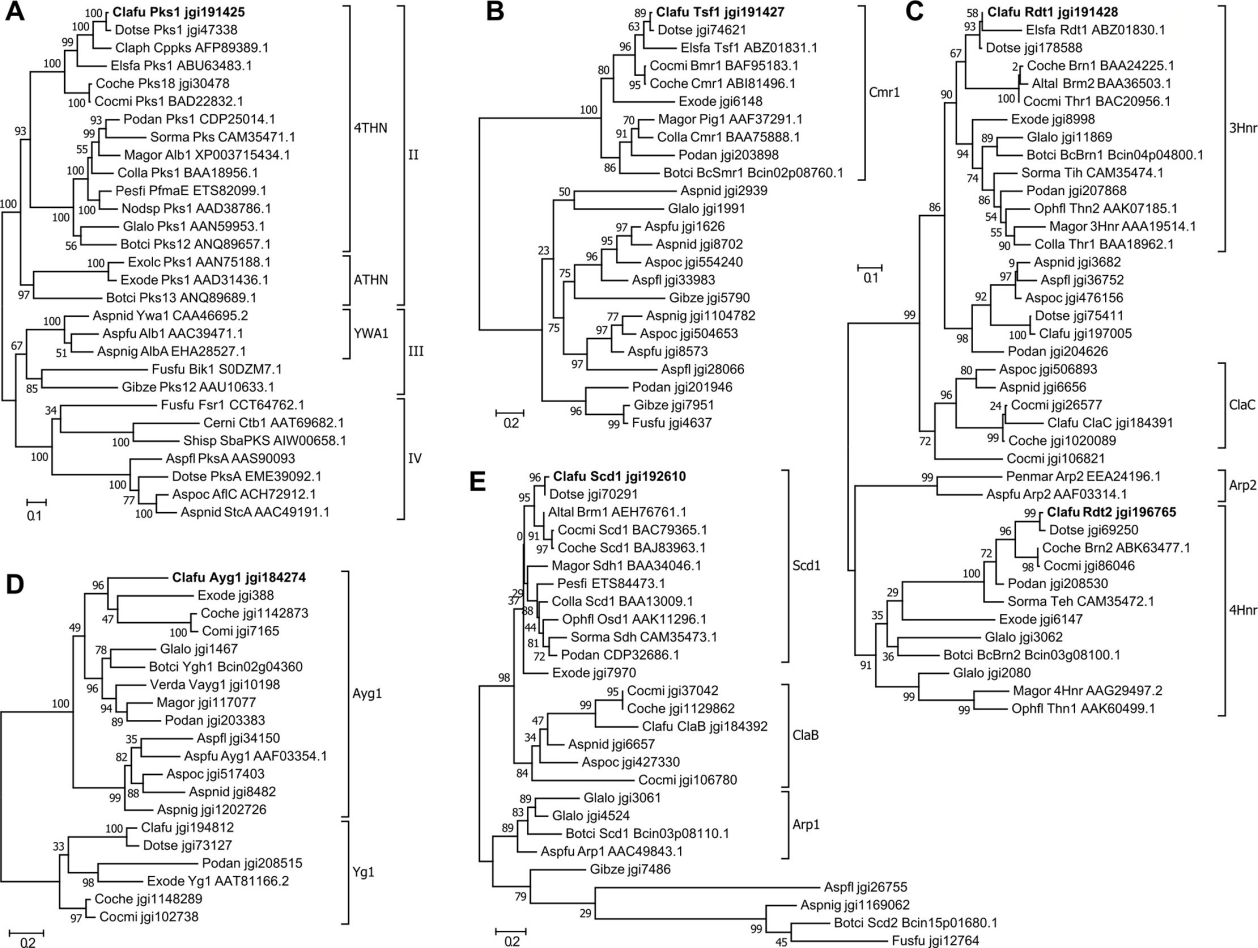
Phylogenetic analysis of proteins involved in DHN melanin biosynthesis. Maximum likelihood phylogenetic trees were built to resolve the evolutionary relationship of **(A)** CfPks1 to characterized non-reducing polyketide synthases; **(B)** CfTsf1 to characterized regulators of DHN melanin biosynthesis; **(C)** CfRdt1 and CfRdt2 to reductases involved in the reduction of 1,3,8-trihydroxy- and 1,3,6,8-tetrahydroxy-naphthalene, respectively; **(D)** CfAyg1 to characterized hydrolases involved in polyketide deacetylation; and **(E)** CfScd1 to characterized scytalone dehydratases. *Elsinoe fawcettii* proteins assigned to the elsinochrome gene cluster are included [41]. Non-characterized homologues are also included for species in which the polyketide synthase involved in DHN synthesis is characterized. The name of characterized proteins and accession numbers (GenBank, SwissProt or Joint Genome Institute (JGI) protein ids) are indicated next to the species acronym. Clafu: *Cladosporium fulvum*; Dotse: *Dothistroma septosporum*; Claph: *Cladosporium phlei*; Elsfa: *Elsinoe fawcettii*; Coche: *Cochliobolus heterostrophus*; Cocmi: *Cochliobolus miyabeanus*; Podan: *Podospora anserina*; Sorma: *Sordaria macrospora*; Magor: *Magnaporthe oryzae*; Colla: *Colletotrichum lagenarium*; Pesfi: *Pestalotiopsis fici*; Nodsp: *Nodulisporium sp*.; Glalo: *Glareae lozoyensis*; Botci: *Botrytis cinerea*; Exolc: *Exophiala lecano-cani*; Exode: *Exophiala dermatitidis*; Aspnid: *Aspergillus nidulans*; Aspfu: *Aspergillus fumigatus*; Aspnig: *Aspergillus niger*; Fusfu: *Fusarium fujikoroi*; Gibze: *Gibberella zeae*; Cerni: *Cercospora nicotianae*; Shisp: *Shiraia sp*.; Aspfl: *Aspergillus flavus*; Aspoc: *Aspergillus ochraroseus*; Altal: *Alternaria alternata*; Ophfl: *Ophiostoma floccosum*; Penmar: *Penicillium marneffei*; Verda: *Verticillium dahliae*.

In a previous study, the *CfPKS1* core gene was found to be highly up regulated in *Δcfwor1* deletion mutants [43]. The published RNA-seq data of this mutant confirms that all genes from the predicted gene cluster are co-regulated, but *CfPRF1* and the predicted border genes are not (Fig 1C). Consistent with previous phylogenetic analyses of 4THN synthases [35,38,45], *CfPKS1*, *CfRDT1* and *CfTSF1* are all orthologous to genes involved in DHN melanin biosynthesis (Figs 2A–2C). In other fungi, this pathway involves three other genes, *AYG1*, *4HNR* and *SCD1* [12,34]. Orthologues of these genes were identified on different scaffolds in the genome of *C*. *fulvum* (Table 1; Figs 2C–2E) and all are significantly up-regulated in the *Δcfwor1* deletion mutants (Fig 1C). In contrast, paralogues of these genes are not differentially expressed or are significantly down-regulated in the *Δcfwor1* deletion mutants (Fig 1C).

**Table 1:** List of *Cladosporium fulvum* genes that are orthologous to melanogenic genes in other fungi.

Gene name	Protein number ^a^	Scaffold	Position	Function	Conserved domains ^b^
*CfPKS1*	191425	scf7180000130411	192,236–198,793	Non-reducing polyketide synthase	PF16073 SAT PF00109/PF02801 KS PF00698 AT PF14765 (dehydratase) PT PF00550 ACP PF00550 ACP PF00975 TE
*CfTSF1*	191427	scf7180000130411	211,934–215,018	Fungal specific transcription factor	pfam00172 Fungal Zn(2)-Cys(6) binuclear cluster domain PF04082 Fungal specific transcription factor domain
*CfRDT1*	191428	scf7180000130411	217,755–218,741	3-hydroxy naphthalene reductase	PF13561 Enoyl-(Acyl carrier protein) reductase
*CfAYG1*	184274	scf7180000126929	44,831–46,097	hydrolase	PF06500 Alpha/beta hydrolase of unknown function
*CfRDT2*	196765	scf7180000130934	32,438–33,312	4-hydroxy naphthalene reductase	PF13561 Enoyl-(Acyl carrier protein) reductase
*CfSCD1*	192610	scf7180000130653	83,529–84,393	Scytalone dehydratase	PF02982 Scytalone dehydratase

^a^ Joint Genome Institute accession number

^b^ Determined using the PFAM database; the typical domains of polyketide synthases are indicated for CfPks1. SAT: Starter unit:ACP transacylase; KS: Ketoacyl Synthase; AT: Acyl Transferase; PT: Product Template; ACP: Acyl Carrier Protein; TE: ThioEsterase. Note that the PT domain is not present in the PFAM database and instead is related to a dehydratase domain.

### CfPks1 is a polyketide synthase that releases 4THN

**Fig 3:**
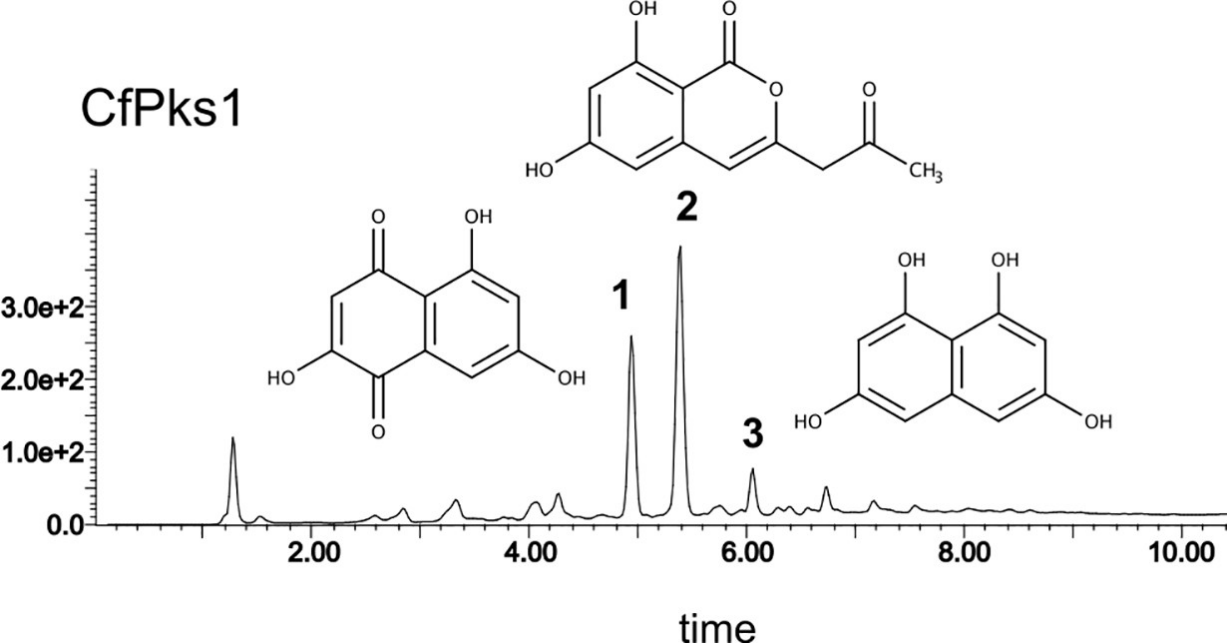
Heterologous expression of CfPks1 in *Aspergillus oryzae*. Representative diode array chromatogram of ethyl acetate extracts from transformants expressing CfPks1. These transformants produced three major compounds that were determined as flaviolin **1**, benzopyran **2** and 1,3,6,8-tetrahydroxy-naphthalene (4THN) **3**.

*CfPKS1* is orthologous to other characterized 4THN synthases from fungi of different orders (Fig 2A). In *C*. *lagenarium*, the 4THN synthase ClPks1 carries a bi-functional TE domain that releases 4THN [18]. CfPks1 was expressed heterologously in *A*. *oryzae* M-2-3 in order to determine whether or not it catalyses the same reactions as ClPks1. Ethyl acetate extracts of transformants contained three major products, **1–3**, bearing UV signatures diagnostic of aromatic polyketides (Fig 3). Product **1** (RT = 4.9 min; UV max = 210, 261, 307 nm; *m/z* (ES^-^) 205 [M-H]^-^) was identified as flaviolin by comparing its UV and mass spectra to published data (S3 and S4 Figs) and was confirmed by High-Resolution Mass Spectrometry (HRMS; exact mass 207.0283; S5A Fig). Flaviolin is a spontaneously oxidised degradation product of 4THN. Product **2** (RT = 5.4 min; UV max = 244, 327 nm; *m/z* (ES^-^) 233 [M-H]^-^) harbours the same chemical formula as benzopyran according to HRMS (exact mass 235.0600; S5B Fig), which is a compound known as a shunt metabolite of the 4THN hexaketide pathway [18]. Product **3** (RT = 6 min; UV max = 244, 325 nm; *m/z* (ES^-^) 191 [M-H]^-^) was identified as 4THN by HRMS (exact mass 193.0498; S5C Fig). These results clearly show that CfPks1 produces the same intermediate as ClPks1 and thus exhibits the same enzymatic activity.

LC-MS analyses of organic extracts obtained from *OE*.*CfTSF1* transformant after 48h growth on induction medium did not detect any precursor of DHN melanin. This observation could be due to a delay between the transcriptional induction and production of the compounds in significant amount as there was no pigmentation difference with wild type. Alternatively, it could suggest that DHN is efficiently polymerized, which is not easily extractable from cell walls with regular chemical methods.

### *CfPKS1* is needed for proper pigmentation of *C*. *fulvum*, but it is not required for pathogenicity on tomato

**Fig 4:**
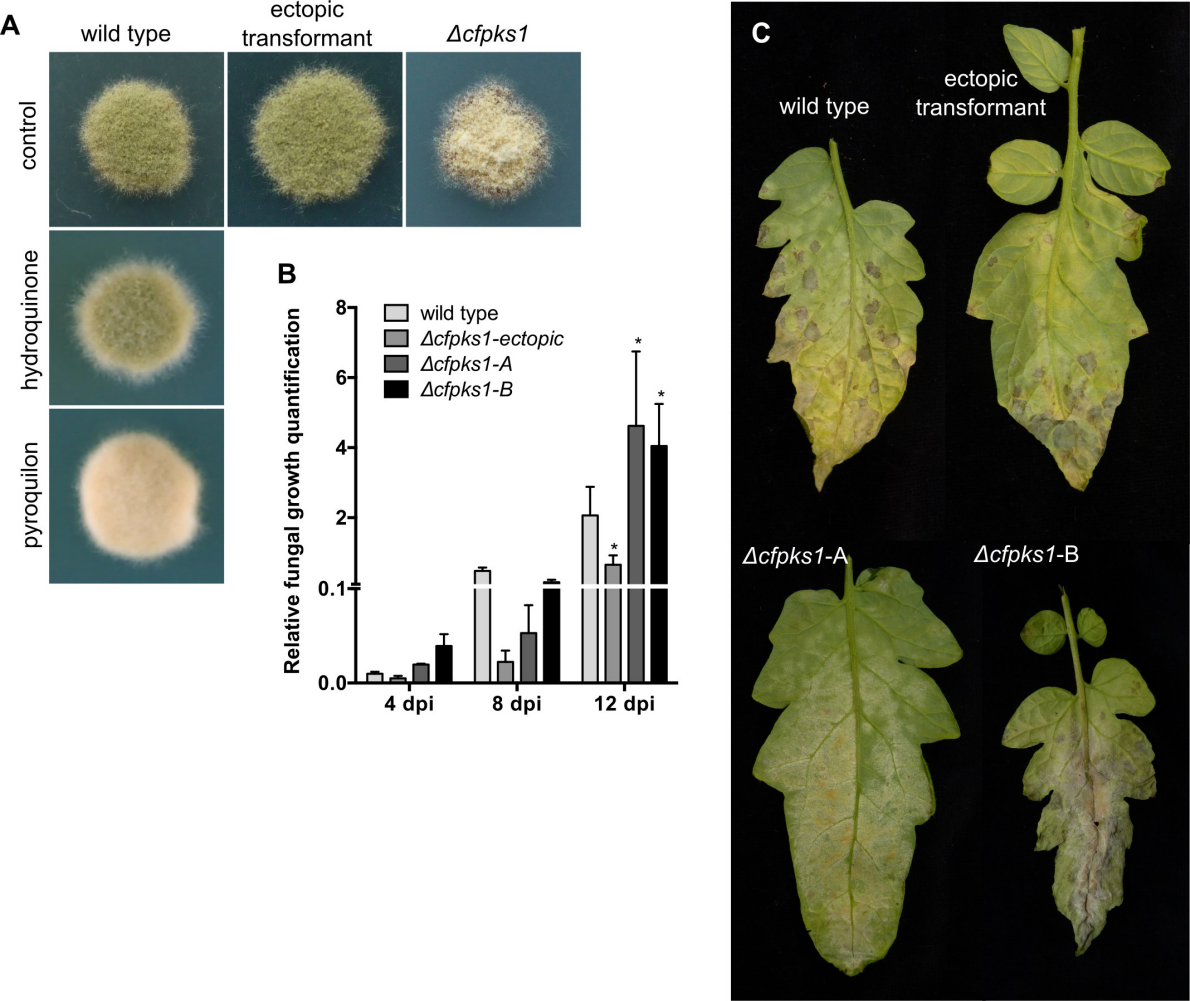
Characterization of *Cladosporium fulvum Δcfpks1* deletion mutants. **(A)** *In vitro* growth of wild-type *C*. *fulvum*, ectopic transformant and *Δcfpks1* deletion mutants on Potato Dextrose Agar (PDA) medium; and wild-type *C*. *fulvum* on PDA supplemented with 30 mg.L^-1^ DHN biosynthesis inhibitor (pyroquilon) or DOPA melanin biosynthesis inhibitor (hydroquinone) [7]. **(B)** Quantification of fungal growth during tomato infection by wild type, an ectopic transformant and *Δcfpks1* deletion mutants. **(C)** Tomato leaves infected by wild type, an ectopic transformant and *Δcfpks1* deletion mutants at 16 days post-inoculation (dpi).

To confirm the heterologous expression results and obtain further insights in the biological role of the *CfPKS1* gene cluster, the *CfPKS1* gene was replaced by a deletion cassette containing the hygromycin resistance marker gene *via* homologous recombination (S1 Fig). Two confirmed independent deletion mutants and an ectopic transformant were selected for further analysis. Both *Δcfpks1* deletion mutants were yellow-orange compared to the grey-green ectopic transformant and wild type (Fig 4A). The wild-type strain shows similar coloration to the *Δcfpks1* deletion mutants when it is grown in the presence of pyroquilon, an inhibitor of the DHN melanin pathway (Fig 4A; [7]). In contrast, it was not coloured differently when grown in the presence of hydroquinone, an inhibitor of DOPA melanin (Fig 4A; [7]). These results contrast with the situation in *D*. *septosporum*, a close relative species of *C*. *fulvum* that produces DOPA melanin despite the presence of *DsPKS1*, the orthologue of *CfPKS1* and other 4THN synthase genes [35]. Together, these results show that the polymerization of DHN melanin contributes to the pigmentation of *C*. *fulvum*. The *Δ*cf*pks1* mutants do not manifest any other obvious developmental or physiological defects.

Because DHN melanin is a pathogenicity factor in several plant pathogens, we addressed the possibility that this role was also true in *C*. *fulvum*. Tomato plants were inoculated with wild-type *C*. *fulvum*, an ectopic transformant and two independent *Δcfpks1* deletion mutants. Whilst the growth of all strains was similar at 4 and 8 dpi, the *Δcfpks1* deletion mutants had significantly outgrown the control strains by 12 dpi (Fig 4B). By 16 dpi, this difference in growth became strikingly clear, as the white-orange deletion mutants had colonized a greater leaf surface area than the control strains (Fig 4C).

## Discussion

### The *CfPKS1* gene cluster is involved in DHN melanin biosynthesis

A previous phylogenetic analysis suggested that *CfPKS1* is an orthologue of *EfPKS1*, which encodes the nrPKS required for elsinochrome production [35,38,42]. The predicted elsinochrome gene cluster is also present in *C*. *fulvum*, except for the putative transporter *ECT1* [38,41]. However, the same phylogenetic analysis showed that *CfPKS1* and *EfPK1* are orthologous to 4THN synthases involved in the biosynthesis of DHN melanin. Similar observations were reported in other phylogenetic analyses [35,45]. Given that PKSs from a monophyletic clade tend to produce the same products, the different chemical structures of DHN melanin and elsinochrome contradict the phylogeny of their respective nrPKSs. The orthologous 4THN synthase in *C*. *lagenarium* has been characterized in detail; ClPks1 synthesizes the hexaketide 2-acetyl-1,3,6,8-tetrahydroxynaphthalene (ATHN) that is cyclized and deacylated by its bi-functional TE domain to give 4THN [18]. In this report, we showed that *C*. *fulvum* CfPks1 is also a synthase that releases 4THN because it produced both the hexaketide benzopyran and the pentaketide 4THN (Fig 3). The precursor ATHN was not detected in our experiments, which might be due to the presence of an expressed homologue of the hydrolase Ayg1 in *A*. *oryzae* that would efficiently deacetylate ATHN to yield 4THN [18, 20].

In characterized nrPKSs involved in DHN melanin biosynthesis, aromatisation of the nascent ACP-bound polyketide to give a monocyclic intermediate is catalysed by the product template (PT) domain [14,16,18,46]. Closure of the second ring and release of the polyketide requires the Claisen/Dieckmann cyclase class of TE domains (TE/CLC) [14,18,46]. The absence of a functional TE domain results in the release of pyrone shunt products *via* spontaneous O-C cyclization [18]. The co-detection of 4THN and benzopyran in *A*. *oryzae*::*CfPKS1* suggests that the TE domain of *CfPKS1* was not fully functional, which might be due to the absence of tailoring enzymes.

Ayg1 and its orthologues were shown to be involved in the deacetylation of a hexaketide or heptaketide precursor in the DHN melanin pathway described in blue/green fungi [24]. However, it was recently shown in the plant pathogen *V*. *dahliae* that *AYG1* might also be important for DHN biosynthesis in brown/black fungi [47]. Our finding that *CfAYG1* is present in *C*. *fulvum* genome and co-regulated with *CfPKS1* suggests that this gene also plays a role in the DHN biosynthetic pathway in *C*. *fulvum*, which requires further investigation. Although another paralogue is present in *C*. *fulvum*, it is not co-regulated with *CfPKS1* and thus is unlikely to be involved in 4THN biosynthesis (Figs 1 and 2). It is noteworthy that Yg1 in *E*. *dermatitidis* is actually a paralogue of characterized Ayg1 in other fungal species (Fig 2). The true orthologue of Ayg1 in *E*. *dermatitidis* must also be further investigated to ascertain its involvement in 4THN production.

Our phylogenetic analysis showed that co-regulated genes at the *CfPKS1* locus in *C*. *fulvum* and genes from the predicted elsinochrome gene cluster (*PKS1*, *RDT1* and *TSF1*) in *E*. *fawcettii* are orthologous to genes involved in DHN biosynthesis (Fig 2). A gene cluster for elsinochrome biosynthesis was recently characterized in *Stagonospora nodorum* [45]. This gene cluster was shown to be related to gene clusters involved in the biosynthesis of cercosporin, a compound of the perylenequinone family that is structurally related to elsinochrome [48–50]. Together with our characterization of CfPks1 as a 4THN synthase, these observations suggest that the elsinochrome gene cluster in *E*. *fawcettii* has not been accurately assigned. Instead, a cercosporin-like gene cluster is certainly involved in the production of elsinochrome as reported in *S*. *nodorum*. The fungus *Cladosporium phlei* produces phleichrome, a perylenequinone that is structurally related to elsinochrome and cercosporin [48]. The *C*. *phlei* nrPKS gene *CpPks1*, orthologous to *CfPKS1* and *EfPKS1*, was assigned to phleichrome production [51]. Similarly, we suggest that *CpPks1* is involved in DHN melanin production and another cercosporin-like nrPKS is responsible for phleichrome production. Such false assignments suggest that crosstalk and interdependencies between gene clusters might be more important than previously thought [52].

### DHN melanin is not a virulence factor in *C*. *fulvum*

The biosynthesis of fungal DHN melanin has been extensively studied because of its diverse roles in fungal biology [15]. Melanin contributes to virulence in animal and plant pathogens, with the latter linked to the formation of host-invading appressoria [15]. In species that do not produce appressoria, melanin confers resilience to chemical and abiotic stresses [12]. *C*. *fulvum* does not differentiate appressoria, which likely explains why DHN melanin is not involved in the pathogenicity of this fungus. The pigmentation of *C*. *fulvum* relies on two compounds, the greyish DHN melanin and yellow-orange-purple (depending on pH) cladofulvin [36,40]. Cladofulvin is also not produced during plant infection, but instead protects the fungus from environmental stresses such as UV light and cold temperatures [37]. Although untested, DHN melanin likely plays a similar role in the survival of *C*. *fulvum ex planta*.

Although further investigation is required to fully exclude that the expression cassette has inserted within a pathogenicity gene, the observed loss of pathogenicity of the *OE*.*CfTSF1* transformant suggests that adequate regulation and downregulation of DHN melanin production during leaf colonization is required for full pathogenicity in *C*. *fulvum*. In *A*. *fumigatus* and *A*. *nidulans*, melanin biosynthesis is initiated in endosomes that carry the enzymes that produce DHN, which is then polymerized within the cell wall by multicopper oxidase and laccase enzymes [53]. Melanin is polymerised in the cell wall, forming several layers of globular particles that grow thicker over time, strengthening the cell wall [54]. It is accepted that hyphal tip elongation requires enzymes to weaken the cell wall in order to incorporate new components, which are then cross-linked to rigidify the cell wall [55]. Thus, the abnormal accumulation of melanin in fungal cell walls is likely to modify its physical properties and increases its rigidity. We observed that the runner hyphae of *C*. *fulvum OE*.*TSF1* transformants branched infrequently and the few successful penetration events resulted in colonizing hyphae that rapidly stopped growing (S2 Fig). This phenotype can be explained by the elevated production of DHN melanin and its abnormal accumulation in cell walls, which might reduce sensitivity to chemotactic gradients and then arrest growth of colonizing hyphae by preventing essential fungal tip remodelling [55]. An *in planta* analysis of fungal cell walls would address this hypothesis. Alternatively, DHN melanin accumulating in the cell wall could be recognized by plant cells, leading to the activation of plant defences and resistance. Similar *in planta* activation might be detrimental to the virulence of other plant pathogens irrespective of whether or not DHN melanin is a pathogenicity factor.

## Conclusion

Using complementary approaches (gene expression, phylogeny, heterologous expression), our study confidently assigned the *CfPKS1* gene cluster to DHN melanin in *C*. *fulvum*. It suggests that orthologous gene clusters in other species have been wrongly assigned to toxin production, including elsinochrome in *E*. *fawcettii* and phleichrome in *C*. *phlei*. Further investigations in these fungal species are needed to address this ambiguity and are likely to provide important insights on pathway crosstalk that might lead to incorrect gene cluster assignment.

## Experimental procedures

Most of the methods were performed as described in Griffiths *et al*. (2016) and Griffiths *et al*. (2018) [37,40].

### Fungal strains employed in this study

*C*. *fulvum* 0WU [39] was the parental strain used to perform transformation and gene deletion experiments. *C*. *fulvum* was grown on potato dextrose agar (PDA) plates at 20°C in the dark. For inhibitor experiments, PDA medium was supplemented with 30 mg.L^-1^ pyroquilon or hydroquinone (Sigma-Aldrich, Zwijndrecht, The Netherlands). *A*. *oryzae* M-2-3 strain was used to perform heterologous expression [56].

### Phylogeny

The protein sequence of CfPks1 and of selected characterized nrPKSs from groups II, III and IV [57] were aligned using Muscle [58] and poorly aligned regions of the alignment were removed using Gblocks, with half allowed gap positions for Ayg1, Rdt and Scd1 alignments, and allowing all gapped positions for the Tsf1 alignment [59]. Maximum-likelihood phylogeny was calculated using PhyML 3.1 [60] with the LG+G+I substitution model as determined by Modelgenerator v851 [61] and SH approximate likelihood ratio test to evaluate branch support.

Non-characterized homologues were retrieved from the Joint Genome Institute MycoCosm portal (genome.jgi.doe.gov; [62]) using BlastP (with default parameters) [63]. All Homologues of 4Hnr (AAG29497.2) and Sdh1 (BAA34046.1) from *M*. *oryzae* and of Ayg1 (AAF03354.1) from *A*. *fumigatus* were sought in the predicted proteome of *C*. *fulvum*. Homologues of CfRdt1, CfTsf1, CfRdt2, CfScd1 and CfAyg1 were sought in selected genomes. For each protein, retrieved homologues and characterized proteins were aligned using Muscle; sequences with large deletions or insertions were manually removed. Neighbour-Joining phylogenetic trees were built with the JTT substitution model using MEGA 7 [64] in order to ascertain orthology. Identified orthologues were then aligned again and analysed following the same process as for CfPks1 described above, but using the LG+G substitution model for Ayg1 and Scd1 trees.

### Generation of *OE*.*CfTSF1* and *OE*.*CfTSF1*::*GFP* transformants

The putative local regulator from the *CfPKS1* gene cluster, *CfTSF1*, was amplified by PCR using Phusion Flash High-Fidelity PCR Master Mix (Life Technologies, Carlsbad, CA) from *C*. *fulvum* genomic DNA using the primer pair *PacI_CfTSF1_Forward* and *NotI_CfTSF1_Reverse* (S1 Table). Plasmid *pFBTS3* contains the promoter of the nitrogen-regulated *Avr9* gene [36,44]. The *CfTSF1* amplicon and *pFBTS3* were cut using *Pac*I and *Not*I restriction enzymes (Fermentas Fast Digest, Waltham, MA), cleaned with Zymogen DNA Clean & Concentrator (Baseclear, Leiden, The Netherlands), and ligated using T4 DNA polymerase (Promega, Madison, WI) to yield *pFBTS3-CfTSF1*. *Escherichia coli* DH5α cells were transformed with the ligation mix using a standard heat-shock protocol and transformants were selected in lysogeny broth (LB)-kanamycin agar (50 μg.ml^-1^). Plasmids were extracted from transformants and screened by restriction digest analysis using *Pac*I and *Not*I in a double digestion. A plasmid bearing the correct restriction pattern was sent to Macrogen (Amsterdam, The Netherlands) for sequencing of the insert. *Agrobacterium tumefaciens* AGL1 was transformed with *pFBTS3-CfTSF1* by electroporation, and plated on LB-kanamycin agar (50 μg.ml^-1^). One positive transformant was picked, verified and named *AT-pFBTS3-CfTSF1*. This plasmid was introduced to *C*. *fulvum* using *A*. *tumefaciens*-mediated transformation as previously described [65]. Transformants were selected on PDA medium supplemented with hygromycin (100 μg.ml^-1^). Several transformants and wild-type *C*. *fulvum* were grown for 5 days in potato-dextrose broth (PDB; Oxoid, Altrincham, UK) and then transferred to Gamborg B5 medium without nitrogen (B5-N) in order to induce the *Avr9* promoter [37,44]. Total RNA was extracted and cDNA synthesis was performed as previously described [65]. The induction of the *CfPKS1* biosynthetic gene cluster was confirmed by RT-qrtPCR using primers listed in S1 Table. One transformant showing the expected strong induction of genes at the *CfPKS1* locus was selected and named *OE*.*CfTSF1*.

Using the same methods, *A*. *tumefaciens* AGL1 was transformed with plasmid *pRM254*, which contains *GFP* and geneticin-resistance genes [66] to yield *AT-pRM254* strain. The plasmid was introduced into the *OE*.*CfTSF1* transformant as described above. Transformants were selected on PDA medium supplemented with geneticin (100 μg.ml^-1^). Transformants were picked and screened for *GFP* fluorescence. One transformant was selected and named *OE*.*CfTSF1*::*GFP*.

### Generation of *Δcfpks1* deletion mutants

The plasmid for targeted gene replacement of *CfPKS1* was generated following the same procedure as described in Griffiths *et al*. (2016) [40]. The upstream (US) and downstream (DS) regions flanking of *CfPKS1* were amplified using primers 1 + 2 and 3 + 4, respectively (S1 Table), and cloned into *pDONR-P4-P1R* and *pDONR-P2-P3* vectors. The final gene replacement plasmid was assembled in a LR reaction (Invitrogen) that combined the *pDONR-P4-P1R*::*US_CfPKS1*, *pDONR-P2-P3*::*DS_CfPKS1*, *p221_GFP_HYG* (*pDONR* containing a cassette with *GFP* and *HYG* resistance marker genes) and the destination vector *pDEST R4-R3* [40,66]. One correct sequenced plasmid was chosen and named *pDest43-****Δ****cfpks1*. This plasmid was introduced into *C*. *fulvum* 0WU using the *A*. *tumefaciens* transformation method as described in Okmen *et al*. (2013) [65]. Transformants were selected on PDA plates containing hygromycin (100 μg.mL^-1^). Genomic DNA of each strain was isolated using a Zymo Research Genomic DNA Clean & Concentrator^TM^ (Baseclear), according to the manufacturer’s recommendations. PCR and quantitative real-time PCR were performed to screen for double crossovers and measure the number of inserted deletion cassettes, respectively (S1 Fig and S1 Table).

### Plant inoculation and determination of fungal growth

Inoculation of tomato with *C*. *fulvum* wild-type, deletion mutant and transformant strains was carried out according to a previously described method [67]. To determine fungal growth, the fourth composite leaf of infected tomato plants was harvested at 4, 8, and 12 days post-inoculation (dpi) and flash frozen in liquid nitrogen. Samples were ground to a fine powder in liquid nitrogen, and total RNA was extracted from 100 mg of material using a Zymogen Direct-zol RNA MiniPrep kit (Baseclear) according to the manufacturer’s recommended protocol. cDNA synthesis was performed using 100–2,000 ng of total RNA and M-MLV reverse transcriptase (Promega), following the manufacturer's protocol. To assess *C*. *fulvum* growth during infection, the *actin* gene of this fungus was targeted by qrtPCR using the *Cf-actin_RT-qrtPCR_F/Cf-actin_RT-qrtPCR_R* primer pair method [67]. For sample calibration, the *Solanum lycopersicum actin* gene was targeted using the *Sl-actin_qrtPCR_F/Sl-actin_qrtPCR_R* primer pair method [67]. The same cDNA samples were used to measure the expression of genes at the *CfPKS1* locus by qrtPCR using previously reported methods and primers [38]. Additional oligonucleotides (S1 Table) were designed and their efficiency determined as described in [38]. Results were analyzed according to the 2^–^***Δ***^Ct^ method [68] and are the average of three biological replicates.

### Microscopic examination of *GFP-*expressing strains

Imaging of infected tomato leaves was performed using a spinning disc confocal microscope (Nikon Ti microscope body (Shinagawa, Tokyo, Japan), Yokogawa CSUX1 scanner (Musashino, Tokyo, Japan), Photometrics Evolve camera (Tucson, AZ), Metamorph software (Molecular Devices, Sunnyvale, CA), 491 nm laser line; 60x oil 1.40NA objective). Z-stacks were acquired with an internal spacing of 0.5 μm. All images were processed using Fiji software [69].

### Construction of vectors for heterologous expression and generation of *A*. *oryzae* M-2-3 transformants

The cloning of *CfPKS1* in heterologous expression vectors was performed as described in Griffiths *et al*. (2016) [40]. Briefly, *CfPKS1* was amplified from *C*. *fulvum OE*.*CfTSF1* transformant cDNA (grown on B5-N medium) by PCR using primers 5 + 6 (S1 Table) and cloned into *Not*I-linearized *pEYA2* using recombination in *S*. *cerevisiae* BMA 64 to generate plasmid *pEYA2-CfPKS1* [40,53]. *CfPKS1* was transferred into the expression vector *pTAex3GS* using LR clonase (Invitrogen), and the resulting *pTAex3GS-CfPKS1* plasmid was introduced in *A*. *oryzae* M-2-3 using PEG-mediated transformation as described in Griffiths *et al*. (2016) [40]. The starch-inducible taka-amylase promoter (*PamyB*) controls the expression of *CfPKS1*. The final vector contains the arginine biosynthesis gene (*argB*) for selection of fungal transformants.

### Secondary metabolite extraction and analysis by LC-MS

Selected *A*. *oryzae* transformants containing *CfPKS1* were grown on dextrose-peptone-yeast extract (DPY) agar plates at 30°C until the whole plates were covered. The cultures were freeze-dried and then homogenised with a pestle and mortar. The homogenate was resuspended in water, acidified to pH4 with HCl, and then twice extracted with ethyl acetate. The organic phase was recovered and dried under a nitrogen flow. Samples were resuspended in acetonitrile (CH_3_CN), centrifuged at 20,000 x g for 5 min in a microcentrifuge tube and then transferred to a 1 mL clear glass shell vial (WAT025054c).

HPLC analysis with a Waters Symmetry reverse phase 5μm, C18, 100 Å column (WAT046980) was carried out on a Waters 600S system. The sample was eluted with a variable gradient of solvents (A) H_2_O and (B) CH_3_CN (both containing 0.1% trifluoroacetic acid) at a flow rate of 1 mL.min^-1^. The following gradient was used: 0 min, A (95%); 10 min, A (10%); 12 min, A (10%), 15 min, A (0%), 16 min, A (95%), 20 min, A (95%). UV spectra were obtained using a 996-photodiode array (PDA) detector and analysed with the Waters Empower software.

LC-MS data were obtained using a Waters LC-MS system composed of a Waters 2767 autosampler, Waters 2545 pump system, a Phenomenex Kinetex column (2.6 μm, C18, 100 Å, 4.6 × 100 mm) equipped with a Phenomenex Security Guard precolumn (Luna C5 300 Å) eluted at 1 mL.min^-1^. Detection was by Waters 2998 Diode Array detector between 200 and 400 nm and Waters SQD-2 mass detector operating simultaneously in ES+ and ES- modes between 100 *m/z* and 650 *m/z*. Solvents were: A, HPLC grade H_2_O containing 0.05% formic acid; B, HPLC grade MeOH containing 0.045% formic acid; and C, HPLC grade CH_3_CN containing 0.045% formic acid). Gradients were as follows: Kinetex/ CH_3_CN: 0 min, 10% C; 10 min, 90% C; 12 min, 90% C; 13 min, 10% C; 15 min, 10% C. Samples were generally diluted to 1 mg.mL^-1^ and 10 μL injected (10 μg). Data capture and analysis, including peak integration, was performed using MassLynx 4.1 software (Waters).

### Semi-preparative LC-MS, compound purification and structure determination

Purification of compounds was achieved using a Waters mass-directed autopurification system comprising a Waters 2767 autosampler, Waters 2545 pump system, a Phenomenex Kinetex Axia column (5 μm, C18, 100 Å, 21.2 × 250 mm) equipped with a Phenomenex Security Guard precolumn (Luna C5 300 Å) eluted at 20 mL.min^-1^ at ambient temperature. Solvent A, HPLC grade H_2_O + 0.05% formic acid; Solvent B, HPLC grade CH_3_CN + 0.045% formic acid. The post-column flow was split (100:1) and the minority flow was made up with HPLC grade MeOH + 0.045% formic acid to 1 mL.min^-1^ for simultaneous analysis by diode array (Waters 2998) and ESI mass spectrometry in positive and negative modes (Waters SQD- 2). Detected peaks were collected into glass test tubes. Combined tubes were evaporated under a flow of dry N_2_ gas and weighed. HRMS data were measured using Waters Q-Tof Premier operating in ES^+^ mode.

## Supporting information

S1 Fig Molecular characterization of *Δcfpks1* deletion mutants and *OE.CfTSF1* transformant. (PNG)

S2 Fig Pathogenicity assay of the *Cladosporium fulvum OE.CfTSF1* transformant and microscopic observation of the OE.*CfTFS1::GFP* over-expression transformant on tomato leaves. (PNG)

S3 Fig Diode array chromatograms (left) and Total Ion Chromatograms (TICs; right) of ethyl acetate extracts from *Aspergillus oryzae* transformants expressing CfPks1.
(PNG)

S4 Fig UV and MS spectra of major products produced by *Aspergillus oryzae* transformants expressing CfPks1. (PNG)

S5 Fig High Resolution Mass Spectrometry (HRMS) data measured for products produced by *Aspergillus oryzae* transformants expressing CfPks1. (PNG)

S1 Table Oligonucleotides used in this study. (DOCX)

## References

[1] Collemare J, Lebrun M-H. Fungal secondary metabolites: ancient toxins and novel effectors in plant–microbe interactions In: Martin F, Kamoun S, editors. Effectors in Plant–Microbe Interactions. Oxford: Wiley-Blackwell; 2011 pp. 377–400.

[2] Stergiopoulos I, Collemare J, Mehrabi R, de Wit PJ. Phytotoxic secondary metabolites and peptides produced by plant pathogenic *Dothideomycete* fungi. FEMS Microbiol. Rev. 2013; 37:67–93. 10.1111/j.1574-6976.2012.00349.x 22931103

[3] Howard RJ, Valent B. Breaking and entering: host penetration by the fungal rice blast pathogen *Magnaporthe grisea*. Annu. Rev. Microbiol. 1996; 50:491–512. 10.1146/annurev.micro.50.1.491 8905089

[4] Chumley F, Valent B. Genetic analysis of melanin-deficient, nonpathogenic mutants of *Magnaporthe grisea*. Mol. Plant-Microbe Interact. 1990; 3:135–143.

[5] Howard RJ, Ferrari MA, Roach DH, Money NP. Penetration of hard substrates by a fungus employing enormous turgor pressures. Proc. Natl. Acad. Sci. USA. 1991; 88:11281–11284. 1837147 10.1073/pnas.88.24.11281 PMC53118

[6] Chen H, Han X, Qin N, Wei L, Yang Y, Rao L, et al Synthesis and biological evaluation of novel inhibitors against 1,3,8-trihydroxynaphthalene reductase from *Magnaporthe grisea*. Bioorg. Med. Chem. 2016; 24:1225–30. 10.1016/j.bmc.2016.01.053 26860927

[7] Lee JK, Jung HM, Kim SY. 1,8-dihydroxynaphthalene (DHN)-melanin biosynthesis inhibitors increase erythritol production in *Torula corallina*, and DHN-melanin inhibits erythrose reductase. Appl. Environ. Microbiol. 2003; 69:3427–34. 10.1128/AEM.69.6.3427-3434.2003 12788746 PMC161539

[8] Woloshuk CP, Sisler HD, Vigil EL. Action of the antipenetrant, tricyclazole, on appressoria of *Pyricularia oryzae*. Physiol. Plant Pathol. 1983; 22:245–259.

[9] Chen Z, Nunes MA, Silva MC, Rodrigues C.J. Jr. Appressorium turgor pressure of *Colletotrichum kahawae* might have a role in coffee cuticle penetration. Mycologia. 2004; 96:1199–1208. 21148942

[10] Gachomo EW, Seufferheld MJ, Kotchoni SO. Melanization of appressoria is critical for the pathogenicity of *Diplocarpon rosae*. Mol. Biol. Rep. 2010; 37:3583–3591. 10.1007/s11033-010-0007-4 20204524

[11] Beltrán-García MJ, Prado FM, Oliveira MS, Ortiz-Mendoza D, Scalfo AC, Pessoa A Jr, et al Singlet molecular oxygen generation by light-activated DHN-melanin of the fungal pathogen *Mycosphaerella fijiensis* in black Sigatoka disease of bananas. PLoS One. 2014; 9:e91616 10.1371/journal.pone.0091616 24646830 PMC3960117

[12] Schumacher J. DHN melanin biosynthesis in the plant pathogenic fungus *Botrytis cinerea* is based on two developmentally regulated key enzyme (PKS)-encoding genes. Mol. Microbiol. 2016; 99:729–48. 10.1111/mmi.13262 26514268

[13] Zhang P, Wang X, Fan A, Zheng Y, Liu X, Wang S, et al A cryptic pigment biosynthetic pathway uncovered by heterologous expression is essential for conidial development in *Pestalotiopsis fici*. Mol Microbiol. 2017; 105:469–483. 10.1111/mmi.13711 28517364

[14] Cox RJ. Polyketides, proteins and genes in fungi: Programmed nano-machines begin to reveal their secrets. Org. Biomol. Chem. 2007; 5:2010–2026. 10.1039/b704420h 17581644

[15] Langfelder K, Streibel M, Jahn B, Haase G, Brakhage AA. Biosynthesis of fungal melanins and their importance for human pathogenic fungi. Fungal Genet. Biol. 2003; 38:143–58. 12620252 10.1016/s1087-1845(02)00526-1

[16] Fujii I, Mori Y, Watanabe A, Kubo Y, Tsuji G, Ebizuka Y. Heterologous expression and product identification of *Colletotrichum lagenarium* polyketide synthase encoded by the *PKS1* gene involved in melanin biosynthesis. Biosci. Biotechnol. Biochem. 1999; 63:1445–52. 10.1271/bbb.63.1445 10501004

[17] Takano Y, Kubo Y, Shimizu K, Mise K, Okuno T, Furusawa I. Structural analysis of PKS1, a polyketide synthase gene involved in melanin biosynthesis in *Colletotrichum lagenarium*. Mol. Gen. Genet. 1995; 249:162–7. 7500937 10.1007/BF00290362

[18] Vagstad AL, Hill EA, Labonte JW, Townsend CA. Characterization of a fungal thioesterase having Claisen cyclase and deacetylase activities in melanin biosynthesis. Chem. Biol. 2012; 19:1525–34. 10.1016/j.chembiol.2012.10.002 23261597 PMC3530136

[19] Feng B, Wang X, Hauser M, Kaufmann S, Jentsch S, Haase G, et al Molecular cloning and characterization of *WdPKS1*, a gene involved in dihydroxynaphthalene melanin biosynthesis and virulence in *Wangiella (Exophiala) dermatitidis*. Infect. Immun. 2001; 69:1781–94. 10.1128/IAI.69.3.1781-1794.2001 11179356 PMC98085

[20] Wheeler MH, Abramczyk D, Puckhaber LS, Naruse M, Ebizuka Y, Fujii I, Szaniszlo PJ. New biosynthetic step in the melanin pathway of *Wangiella* (*Exophiala*) *dermatitidis*: evidence for 2-acetyl-1,3,6,8-Tetrahydroxynaphthalene as a novel precursor. Eukaryot Cell. 2008; 7:1699–711. 10.1128/EC.00179-08 18676950 PMC2568069

[21] Chiang YM, Meyer KM, Praseuth M, Baker SE, Bruno KS, Wang CC. Characterization of a polyketide synthase in *Aspergillus niger* whose product is a precursor for both dihydroxynaphthalene (DHN) melanin and naphtho-γ-pyrone. Fungal Genet. Biol. 2011; 48:430–7. 10.1016/j.fgb.2010.12.001 21176790 PMC3118676

[22] Tsai HF, Chang YC, Washburn RG, Wheeler MH, Kwon-Chung KJ. The developmentally regulated *alb1* gene of *Aspergillus fumigatus*: its role in modulation of conidial morphology and virulence. J. Bacteriol. 1998; 180:3031–8. 9620950 10.1128/jb.180.12.3031-3038.1998 PMC107801

[23] Watanabe A, Fujii I, Tsai H, Chang YC, Kwon-Chung KJ, Ebizuka Y. *Aspergillus fumigatus alb1* encodes naphthopyrone synthase when expressed in *Aspergillus oryzae*. FEMS Microbiol. Lett. 2000; 192:39–44. 10.1111/j.1574-6968.2000.tb09356.x 11040426

[24] Fujii I, Yasuoka Y, Tsai HF, Chang YC, Kwon-Chung KJ, Ebizuka Y. Hydrolytic polyketide shortening by ayg1p, a novel enzyme involved in fungal melanin biosynthesis. J. Biol. Chem. 2004; 279:44613–20. 10.1074/jbc.M406758200 15310761

[25] Tsai HF, Fujii I, Watanabe A, Wheeler MH, Chang YC, Yasuoka Y, et al Pentaketide melanin biosynthesis in *Aspergillus fumigatus* requires chain-length shortening of a heptaketide precursor. J. Biol. Chem. 2001; 276:29292–8. 10.1074/jbc.M101998200 11350964

[26] Thompson JE, Fahnestock S, Farrall L, Liao DI, Valent B, Jordan DB. The second naphthol reductase of fungal melanin biosynthesis in *Magnaporthe grisea*: tetrahydroxynaphthalene reductase. J Biol Chem. 2000; 275:34867–72. 10.1074/jbc.M006659200 10956664

[27] Lin SY, Okuda S, Ikeda K, Okuno T, Takano Y. *LAC2* encoding a secreted laccase is involved in appressorial melanization and conidial pigmentation in *Colletotrichum orbiculare*. Mol. Plant Microbe Interact. 2012; 25:1552–61. 10.1094/MPMI-05-12-0131-R 22934563

[28] Saitoh Y, Izumitsu K, Morita A, Shimizu K, Tanaka C. ChMCO1 of *Cochliobolus heterostrophus* is a new class of metallo-oxidase, playing an important role in DHN-melanization. Mycoscience. 2010; 51:327–336.

[29] Sugareva V, Härtl A, Brock M, Hübner K, Rohde M, Heinekamp T, et al Characterisation of the laccase-encoding gene *abr2* of the dihydroxynaphthalene-like melanin gene cluster of *Aspergillus fumigatus*. Arch. Microbiol. 2006; 186:345–55. 10.1007/s00203-006-0144-2 16988817

[30] Tsai HF, Wheeler MH, Chang YC, Kwon-Chung KJ. A developmentally regulated gene cluster involved in conidial pigment biosynthesis in *Aspergillus fumigatus*. J. Bacteriol. 1999; 181:6469–77. 10515939 10.1128/jb.181.20.6469-6477.1999 PMC103784

[31] Woo PC, Tam EW, Chong KT, Cai JJ, Tung ET, Ngan AH, et al High diversity of polyketide synthase genes and the melanin biosynthesis gene cluster in *Penicillium marneffei*. FEBS J. 2010; 277:3750–8. 10.1111/j.1742-4658.2010.07776.x 20718860

[32] Kimura N., Tsuge T. Gene cluster involved in melanin biosynthesis of the filamentous fungus *Alternaria alternata*. J. Bacteriol. 1993; 175:4427–35. 8392512 10.1128/jb.175.14.4427-4435.1993 PMC204883

[33] Fetzner R, Seither K, Wenderoth M, Herr A, Fischer R. *Alternaria alternata* transcription factor CmrA controls melanization and spore development. Microbiology. 2014; 160:1845–54. 10.1099/mic.0.079046-0 24972701

[34] Teichert I, Nowrousian M. Evolution of genes for secondary metabolism in fungi In: Pöggeler S, Wöstemeyer J, editors. The Mycota XIV, Evolution of fungi and fungal-like organisms. Springer-Verlag Berlin Heidelberg; 2011 pp. 231–255.

[35] Ozturk IK, Chettri P, Dupont PY, Barnes I, McDougal RL, Moore GG, et al Evolution of polyketide synthesis in a *Dothideomycete* forest pathogen. Fungal Genet. Biol. 2017; 106:42–50. 10.1016/j.fgb.2017.07.001 28690095

[36] Griffiths S, Saccomanno B, de Wit PJ, Collemare J. Regulation of secondary metabolite production in the fungal tomato pathogen *Cladosporium fulvum*. Fungal Genet. Biol. 2015; 84:52–61. 10.1016/j.fgb.2015.09.009 26415644

[37] Griffiths S, Mesarich CH, Overdijk EJR, Saccomanno B, de Wit PJGM, Collemare J. Down-regulation of cladofulvin biosynthesis is required for biotrophic growth of *Cladosporium fulvum* on tomato. Mol Plant Pathol. 2018; 19:369–380. 10.1111/mpp.12527 27997759 PMC6638085

[38] Collemare J, Griffiths S, Iida Y, Karimi Jashni M, Battaglia E, Cox RJ, et al Secondary metabolism and biotrophic lifestyle in the tomato pathogen *Cladosporium fulvum*. PLoS One. 2014; 9:e85877 10.1371/journal.pone.0085877 24465762 PMC3895014

[39] de Wit PJ, van der Burgt A, Ökmen B, Stergiopoulos I, Abd-Elsalam KA, Aerts AL, et al The genomes of the fungal plant pathogens *Cladosporium fulvum* and *Dothistroma septosporum* reveal adaptation to different hosts and lifestyles but also signatures of common ancestry. PLoS Genet. 2012; 8:e1003088 10.1371/journal.pgen.1003088 23209441 PMC3510045

[40] Griffiths S, Mesarich CH, Saccomanno B, Vaisberg A, de Wit PJ, Cox R, et al Elucidation of cladofulvin biosynthesis reveals a cytochrome P450 monooxygenase required for anthraquinone dimerization. Proc. Natl. Acad. Sci. USA. 2016; 113:6851–6. 10.1073/pnas.1603528113 27274078 PMC4922171

[41] Chung KR, Liao HL. Determination of a transcriptional regulator-like gene involved in biosynthesis of elsinochrome phytotoxin by the citrus scab fungus, *Elsinoë fawcettii*. Microbiology. 2008; 154:3556–66. 10.1099/mic.0.2008/019414-0 18957608

[42] Liao HL, Chung KR. Genetic dissection defines the roles of elsinochrome phytotoxin for fungal pathogenesis and conidiation of the citrus pathogen *Elsinoë fawcettii*. Mol. Plant Microbe Interact. 2008; 21:469–79. 10.1094/MPMI-21-4-0469 18321192

[43] Ökmen B, Collemare J, Griffiths S, van der Burgt A, Cox R, de Wit PJ. Functional analysis of the conserved transcriptional regulator CfWor1 in *Cladosporium fulvum* reveals diverse roles in the virulence of plant pathogenic fungi. Mol. Microbiol. 2014; 92:10–27. 10.1111/mmi.12535 24521437

[44] van den Ackerveken GF, Dunn RM, Cozijnsen AJ, Vossen JP, van den Broek HW, de Wit PJ. Nitrogen limitation induces expression of the avirulence gene *avr9* in the tomato pathogen *Cladosporium fulvum*. Mol. Gen. Genet. 1994; 243, 277–285. 8190081 10.1007/BF00301063

[45] Chooi YH, Zhang G, Hu J, Muria-Gonzalez MJ, Tran PN, Pettitt A, et al Functional genomics-guided discovery of a light-activated phytotoxin in the wheat pathogen *Parastagonospora nodorum via* pathway activation. Environ. Microbiol. 2017; 19:1975–1986. 10.1111/1462-2920.13711 28251756

[46] Watanabe A, Ebizuka Y. Unprecedented mechanism of chain length determination in fungal aromatic polyketide synthases. Chem. Biol. 2004; 11:1101–6. 10.1016/j.chembiol.2004.05.015 15324811

[47] Fan R, Klosterman SJ, Wang C, Subbarao KV, Xu X, Shang W, et al Vayg1 is required for microsclerotium formation and melanin production in *Verticillium dahliae*. Fungal Genet. Biol. 2017; 98:1–11. 10.1016/j.fgb.2016.11.003 27866941

[48] Daub ME, Herrero S, Chung KR. Photoactivated perylenequinone toxins in fungal pathogenesis of plants. FEMS Microbiol. Lett. 2005; 252:197–206. 10.1016/j.femsle.2005.08.033 16165316

[49] Chen H, Lee MH, Daub ME, Chung KR. Molecular analysis of the cercosporin biosynthetic gene cluster in *Cercospora nicotianae*. Mol. Microbiol. 2007; 64:755–70. 10.1111/j.1365-2958.2007.05689.x 17462021

[50] Newman AG, Townsend CA. Molecular characterization of the cercosporin biosynthetic pathway in the fungal plant pathogen *Cercospora nicotianae*. J. Am. Chem. Soc. 2016; 138:4219–28. 10.1021/jacs.6b00633 26938470 PMC5129747

[51] So KK, Chung YJ, Kim JM, Kim BT, Park SM, Kim DH. Identification of a polyketide synthase gene in the synthesis of phleichrome of the phytopathogenic fungus *Cladosporium phlei*. Mol. Cells. 2015; 38:1105–10. 10.14348/molcells.2015.0208 26612679 PMC4697002

[52] Bergmann S, Funk AN, Scherlach K, Schroeckh V, Shelest E, Horn U, et al Activation of a silent fungal polyketide biosynthesis pathway through regulatory cross talk with a cryptic nonribosomal peptide synthetase gene cluster. Appl. Environ. Microbiol. 2010; 76:8143–9. 10.1128/AEM.00683-10 20952652 PMC3008269

[53] Upadhyay S, Xu X, Lowry D, Jackson JC, Roberson RW, Lin X. Subcellular compartmentalization and trafficking of the biosynthetic machinery for fungal melanin. Cell Rep. 2016; 14:2511–8. 10.1016/j.celrep.2016.02.059 26972005 PMC4805463

[54] Eisenman HC, Nosanchuk JD, Webber JB, Emerson RJ, Camesano TA, Casadevall A. Microstructure of cell wall-associated melanin in the human pathogenic fungus *Cryptococcus neoformans*. Biochemistry. 2005; 44:3683–93. 10.1021/bi047731m 15751945

[55] Riquelme M. Tip growth in filamentous fungi: a road trip to the apex. Annu, Rev. Microbiol. 2013; 67:587–609. 23808332 10.1146/annurev-micro-092412-155652

[56] Pahirulzaman KAK, Williams K, Lazarus CM. A toolkit for heterologous expression of metabolic pathways in *Aspergillus oryzae*. Methods Enzymol. 2012; 517:241–260. 10.1016/B978-0-12-404634-4.00012-7 23084942

[57] Throckmorton K, Wiemann P, Keller NP. Evolution of chemical diversity in a group of non-reduced polyketide gene clusters: using phylogenetics to inform the search for novel fungal natural products. Toxins (Basel). 2015; 7:3572–607. 26378577 10.3390/toxins7093572 PMC4591646

[58] Edgar RC. MUSCLE: multiple sequence alignment with high accuracy and high throughput. Nucleic Acids Research. 2004; 32, 1792–1797. 10.1093/nar/gkh340 15034147 PMC390337

[59] Castresana J. Selection of conserved blocks from multiple alignments for their use in phylogenetic analysis. Mol. Biol. Evol. 2000; 17: 540–552. 10.1093/oxfordjournals.molbev.a026334 10742046

[60] Guindon S, Gascuel O. A simple, fast, and accurate algorithm to estimate large phylogenies by maximum likelihood. Syst. Biol. 2003; 52:696–704. 14530136 10.1080/10635150390235520

[61] Keane TM, Creevey CJ, Pentony MM, Naughton TJ, Mclnerney JO. Assessment of methods for amino acid matrix selection and their use on empirical data shows that ad hoc assumptions for choice of matrix are not justified. BMC Evol. Biol. 2006; 6:29 10.1186/1471-2148-6-29 16563161 PMC1435933

[62] Grigoriev IV, Nikitin R, Haridas S, Kuo A, Ohm R, Otillar R, et al MycoCosm portal: gearing up for 1000 fungal genomes. Nucleic Acids Res. 2014; 42:D699–704. 10.1093/nar/gkt1183 24297253 PMC3965089

[63] Altschul SF, Gish W, Miller W, Myers EW, Lipman DJ. Basic local alignment search tool. J. Mol. Biol. 1990; 215:403–410. 10.1016/S0022-2836(05)80360-2 2231712

[64] Kumar S, Stecher G, Tamura K. MEGA7: Molecular Evolutionary Genetics Analysis version 7.0 for bigger datasets. Mol. Biol. Evol. 2016; 33:1870–4. 10.1093/molbev/msw054 27004904 PMC8210823

[65] Ökmen B, Etalo DW, Joosten MH, Bouwmeester HJ, de Vos RC, Collemare J, et al Detoxification of α-tomatine by *Cladosporium fulvum* is required for full virulence on tomato. New Phytol. 2013; 198:1203–14. 10.1111/nph.12208 23448507

[66] Mehrabi R, Mirzadi Gohari A, da Silva GF, Steinberg G, Kema GH, de Wit PJ. Flexible gateway constructs for functional analyses of genes in plant pathogenic fungi. Fungal Genet. Biol. 2015; 79:186–92. 10.1016/j.fgb.2015.03.016 26092806

[67] Mesarich CH, Griffiths SA, van der Burgt A, Ökmen B, Beenen HG, Etalo DW, et al Transcriptome sequencing uncovers the *Avr5* avirulence gene of the tomato leaf mold pathogen *Cladosporium fulvum*. Mol. Plant Microbe Interact. 2014; 27:846–57. 10.1094/MPMI-02-14-0050-R 24678832

[68] Livak KJ, Schmittgen TD. Analysis of relative gene expression data using real-time quantitative PCR and the 2(–Delta Delta C(T)) method. Methods. 2001; 25, 402–408. 10.1006/meth.2001.1262 11846609

[69] Schindelin J, Arganda-Carreras I, Frise E, Kaynig V, Longair M, Pietzsch T, et al Fiji: an open-source platform for biological-image analysis. Nat. Methods. 2012; 9:676–82. 10.1038/nmeth.2019 22743772 PMC3855844

