# Supplementary figures and images for: Assignment of a dubious gene cluster to melanin biosynthesis in the tomato fungal pathogen Cladosporium fulvum
Source: PLoS One. 2018 Dec 31;13(12):e0209600. doi: 10.1371/journal.pone.0209600 (PMC6312243; doi:10.1371/journal.pone.0209600)

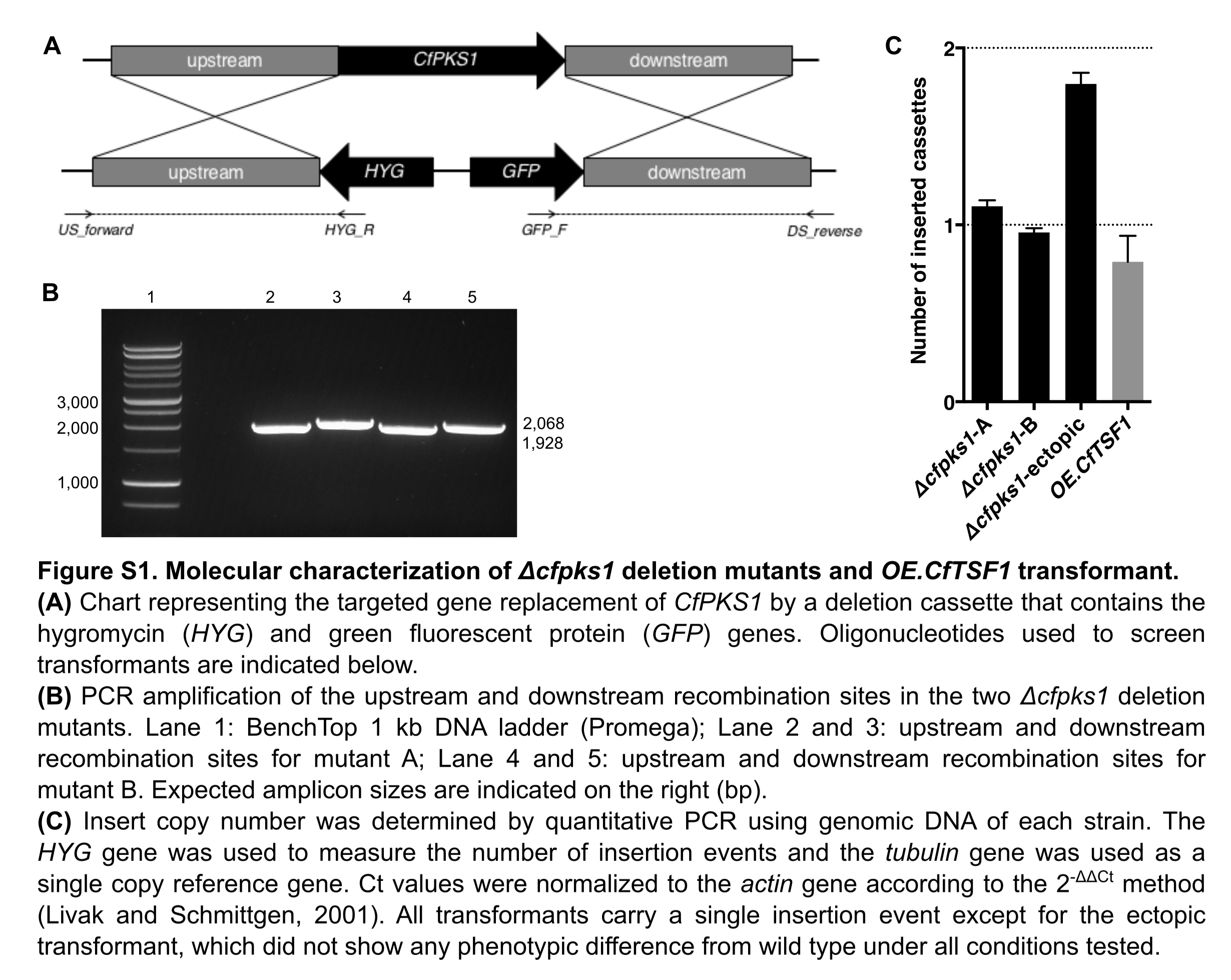

Supplement: S1 Fig — (PNG) [file pone.0209600.s001.png]

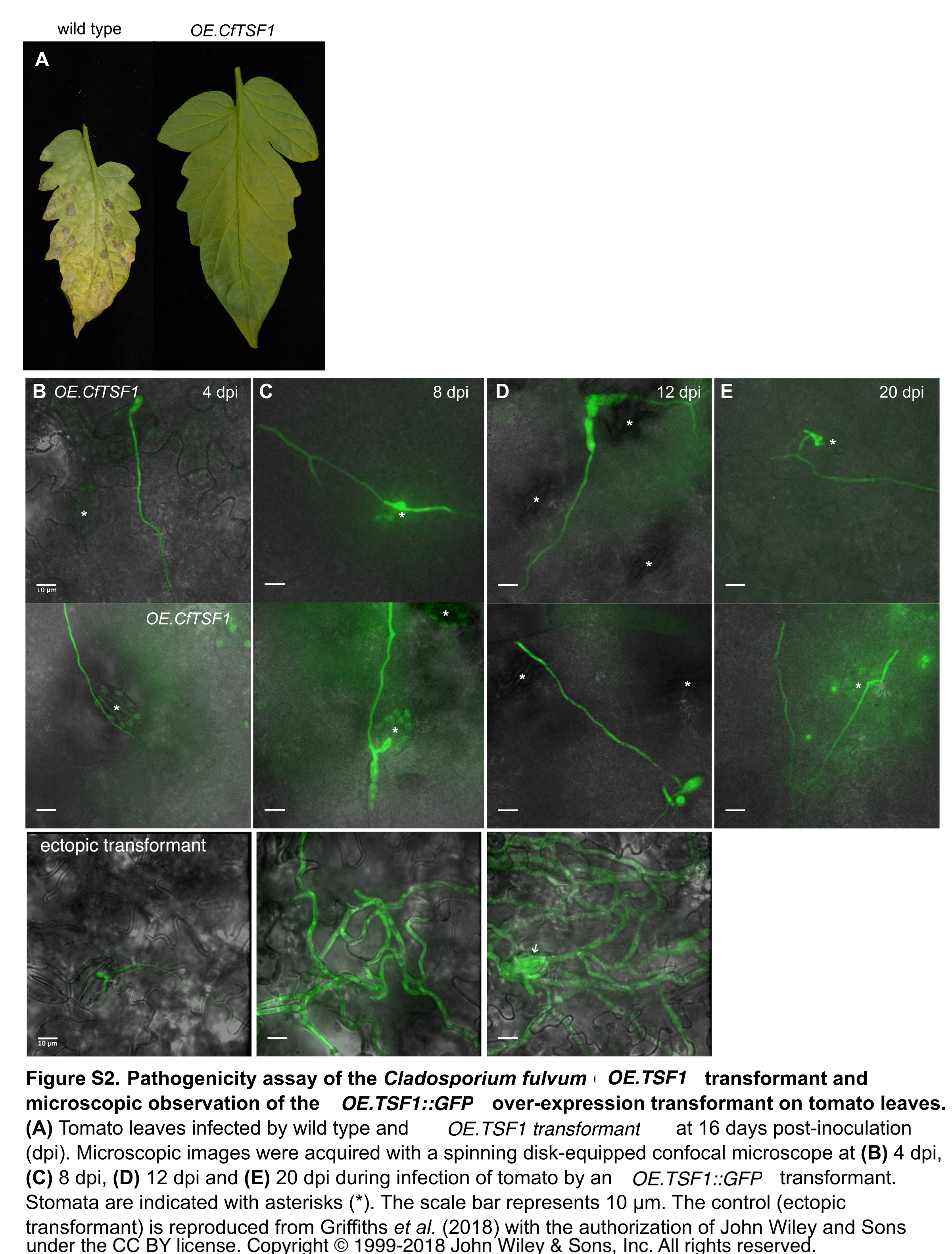

Supplement: S2 Fig — (PNG) [file pone.0209600.s002.png]

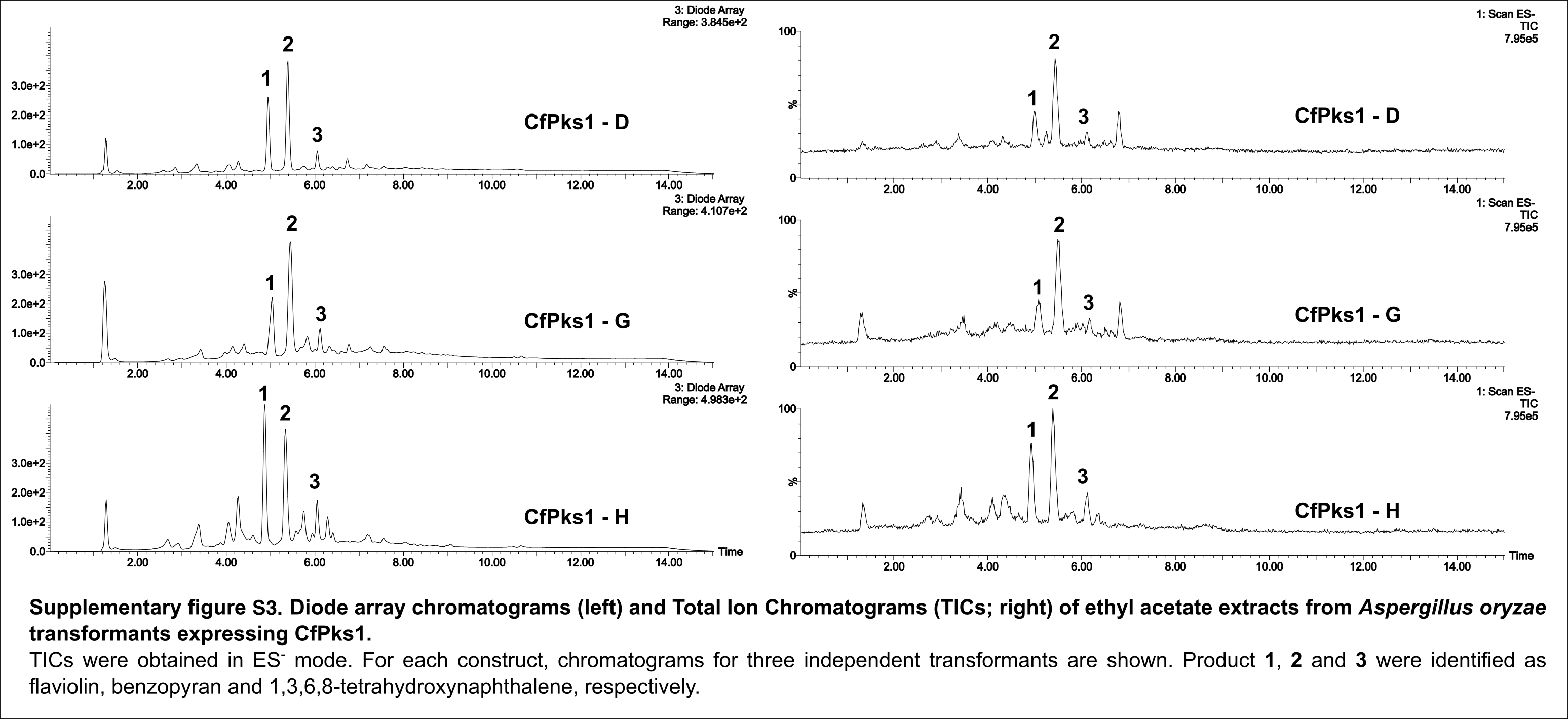

Supplement: S3 Fig — Diode array chromatograms (left) and Total Ion Chromatograms (TICs; right) of ethyl acetate extracts from Aspergillus oryzae transformants expressing CfPks1. (PNG) [file pone.0209600.s003.png]

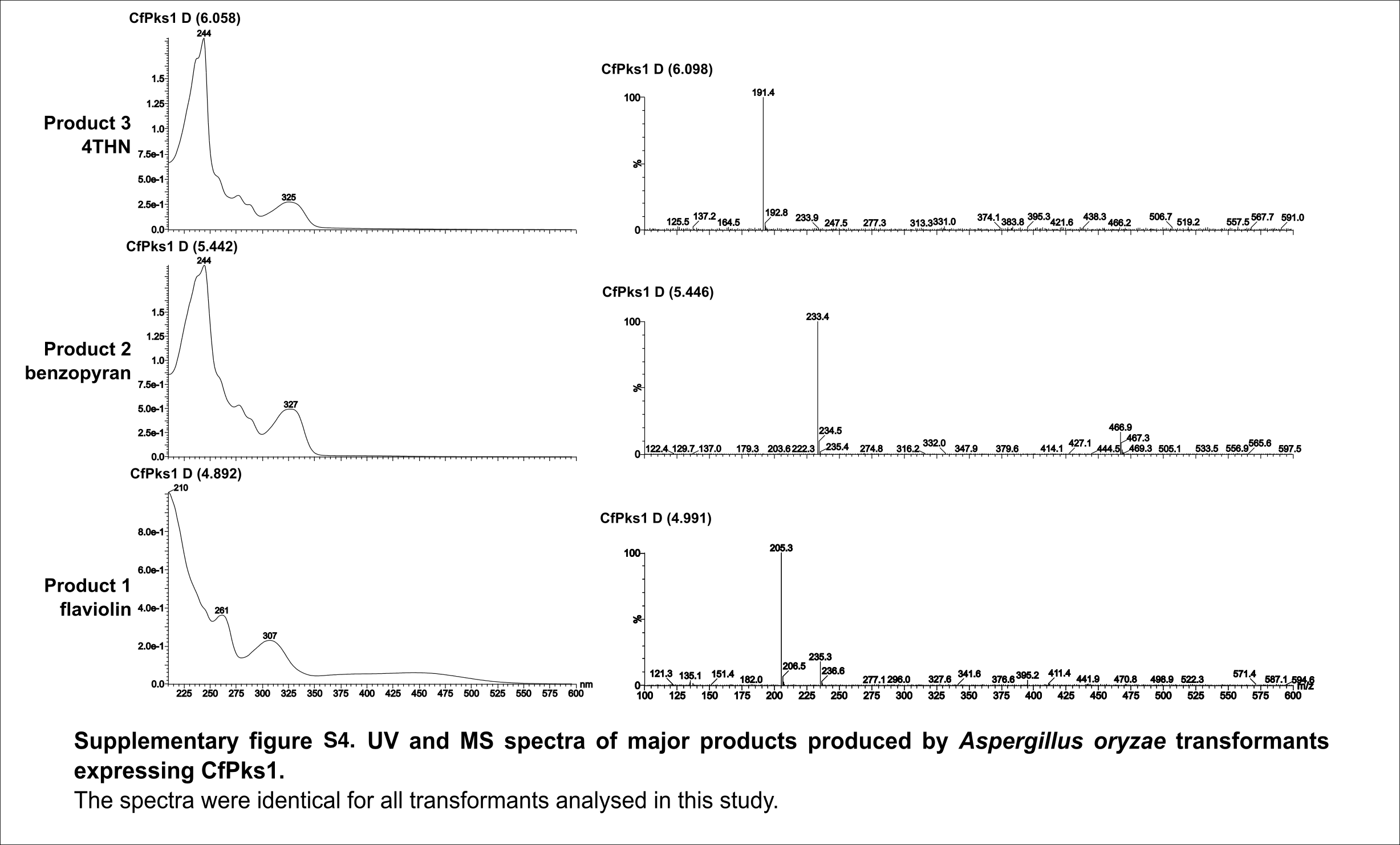

Supplement: S4 Fig — (PNG) [file pone.0209600.s004.png]

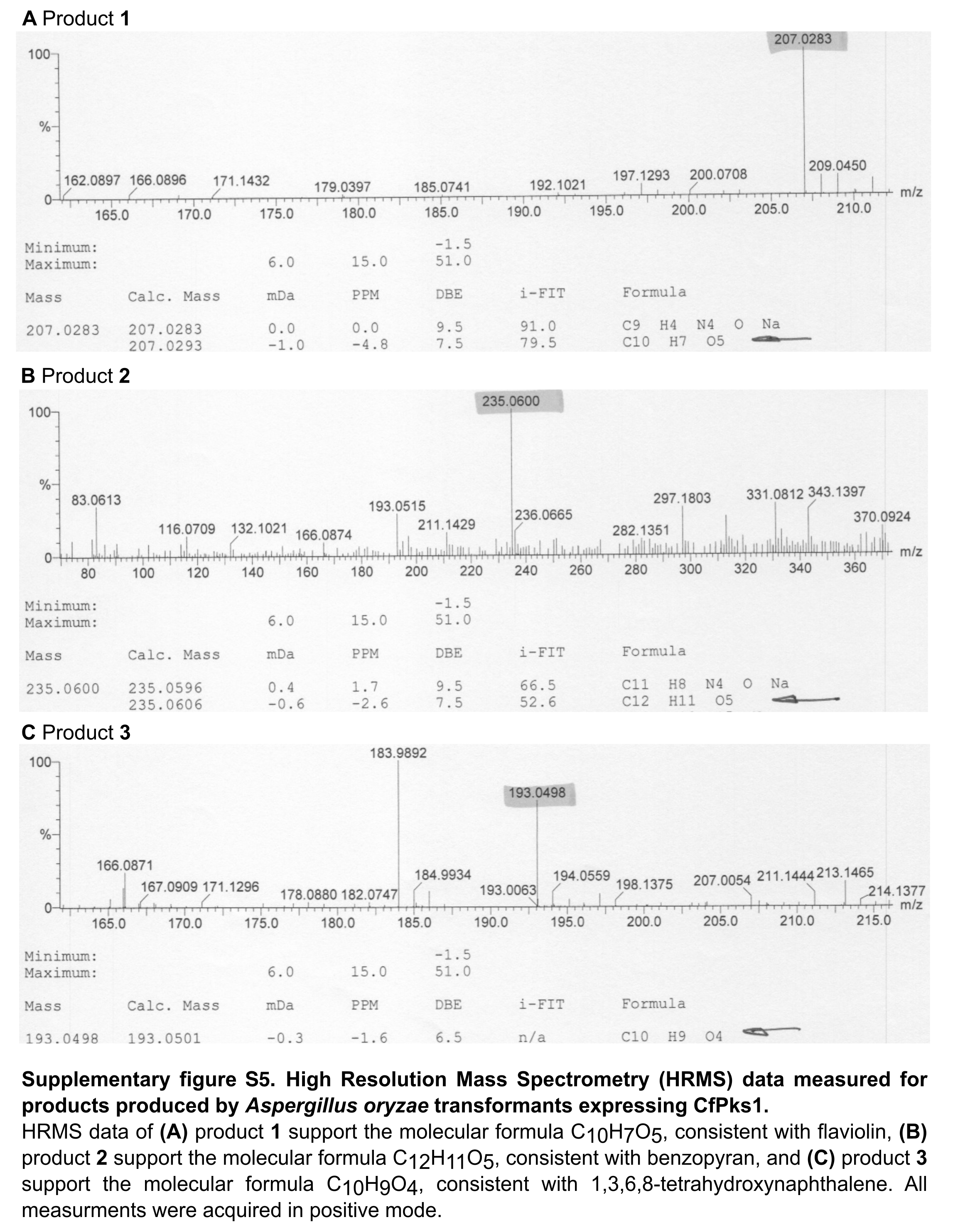

Supplement: S5 Fig — (PNG) [file pone.0209600.s005.png]
