# Supplementary material for: Assignment of a dubious gene cluster to melanin biosynthesis in the tomato fungal pathogen Cladosporium fulvum
Source: PLoS One. 2018 Dec 31;13(12):e0209600. doi: 10.1371/journal.pone.0209600 (PMC6312243; doi:10.1371/journal.pone.0209600)
Supplement: S1 Table — (DOCX) [file pone.0209600.s006.docx]

**S1 Table. Oligonucleotides used in this study.**

| **Name** | | **Sequence (5’ to 3’)** | | **Expected fragment size (bp)** |
| --- | --- | --- | --- | --- |
| **Construction of deletion cassettes** | | | | |
| **1** | *CfPKS1_US_F* | GGGGACAACTTTGTATAGAAAAGTTGTATGGTACCGAACAGCATGG | | 1000 |
| **2** | *CfPKS1_US_R* | GGGGACTGCTTTTTTGTACAAACTTGTTCTTCGGTATTTCGGATCG | |  |
| **3** | *CfPKS1_DS_F* | GGGGACAGCTTTCTTGTACAAAGTGGTCACTTTGGACATCCAGTCG | | 1060 |
| **4** | *CfPKS1_DS_R* | GGGGACAACTTTGTATAATAAAGTTGAGGTCAGCGGTACTGCAACT | |  |
| **Construction of plasmids for heterologous expression** | | | | |
| **5** | *pEYA2::CfPKS1* | TAATGCCAACTTTGTACAAAAAAGCAGGCTATGTCGAACGTTCTGCTTTTCGGCGAC | | 6618 |
| **6** | *CfPKS1::pEYA2* | TAATGCCAACTTTGTACAAGAAAGCTGGGTTCATGCTCTCCAGTCAAGTCCCTC | |  |
| **Verification of transformants** | | | | |
| *Delta PKS1 Verify F* | | GAAGTGCACCAGCGGATGCCTACC | | See Figure S1 |
| *Delta PKS1 Verify R* | | GTGGTCTGCTGTTTATCCGGCCATC | |  |
| *Hyg_Ver_R* | | CACTCGTCCGAGGGCAAAGGAATAG | |  |
| *Gfp_Ver_F* | | cacggcatggacgagctgtacaag | |  |
| **qrtPCR** | | **Forward** | **Reverse** |  |
| *191423* | | CTCAGGCAGGGTTCTCTTTG | GCAGCTCGACCTCCAGTATC |  |
| *191424* (*CfPRF1*) | | GGAGCGAGTCGAGGACTATG | CGCATAGAGCGTATCGTTCA |  |
| *191425* (*CfPKS1*) | | GTGATGCACTGAAGGCTCAA | AGCAAGTTGGTCGAGCTGAT |  |
| *191426* | | GCTGCCACGAGACAACACTA | AGACGATGGGAGTCAAGCAG |  |
| *191427* (*CfTSF1*) | | GCCGTCAAAGTTCTCTCGTC | GCTCGAGGAAGTCCAGTCAC |  |
| *191428* (*CfRDT1*) | | TGTCTACTCCGGATCCAAGG | GGACCATGTCTTGGCGTACT |  |
| *191429* | | GTGAAACGCTTGAGCAAACA | CATTGATGGTTTCGGCTTCT |  |

Annealing temperature for all oligonucleotide pairs is 60°C
